# Allylmethylsulfide, a Sulfur Compound Derived from Garlic, Attenuates Isoproterenol-Induced Cardiac Hypertrophy in Rats

**DOI:** 10.1155/2020/7856318

**Published:** 2020-06-11

**Authors:** Soheb Anwar Mohammed, Bugga Paramesha, Yashwant Kumar, Ubaid Tariq, Sudheer Kumar Arava, Sanjay Kumar Banerjee

**Affiliations:** ^1^Non-Communicable Diseases Group, Translational Health Science and Technology Institute (THSTI), 121001, Faridabad, India; ^2^Department of Pathology, All India Institute of Medical Sciences, New Delhi 110029, India; ^3^Department of Biotechnology, National Institute of Pharmaceutical Education and Research (NIPER), Guwahati, Assam 781101, India

## Abstract

Allylmethylsulfide (AMS) is a novel sulfur metabolite found in the garlic-fed serum of humans and animals. In the present study, we have observed that AMS is safe on chronic administration and has a potential antihypertrophic effect. Chronic administration of AMS for 30 days did not cause any significant differences in the body weight, electrocardiogram, food intake, serum biochemical parameters, and histopathology of vital organs. Single-dose pharmacokinetics of AMS suggests that AMS is rapidly metabolized into Allylmethylsulfoxide (AMSO) and Allylmethylsulfone (AMSO_2_). To evaluate the efficacy of AMS, cardiac hypertrophy was induced by subcutaneous implantation of ALZET® osmotic minipump containing isoproterenol (~5 mg/kg/day), cotreated with AMS (25 and 50 mg/kg/day) and enalapril (10 mg/kg/day) for 2 weeks. AMS and enalapril significantly reduced cardiac hypertrophy as studied by the heart weight to body weight ratio and mRNA expression of fetal genes (ANP and *β*-MHC). We have observed that TBARS, a parameter of lipid peroxidation, was reduced and the antioxidant enzymes (glutathione, catalase, and superoxide dismutase) were improved in the AMS and enalapril-cotreated hypertrophic hearts. The extracellular matrix (ECM) components such as matrix metalloproteinases (MMP2 and MMP9) were significantly upregulated in the diseased hearts; however, with the AMS and enalapril, it was preserved. Similarly, caspases 3, 7, and 9 were upregulated in hypertrophic hearts, and with the AMS and enalapril treatment, they were reduced. Further to corroborate this finding with *in vitro* data, we have checked the nuclear expression of caspase 3/7 in the H9c2 cells treated with isoproterenol and observed that AMS cotreatment reduced it significantly. Histopathological investigation of myocardium suggests AMS and enalapril treatment reduced fibrosis in hypertrophied hearts. Based on our experimental results, we conclude that AMS, an active metabolite of garlic, could reduce isoproterenol-induced cardiac hypertrophy by reducing oxidative stress, apoptosis, and stabilizing ECM components.

## 1. Introduction

Cardiovascular diseases (CVDs) contribute the highest among the noncommunicable disease's deaths globally; nearly 17.8 million deaths were reported due to CVDs alone in the year 2017 [1]. Cardiac hypertrophy (CH) is a compensatory phase of the heart against various underlying pathophysiological stimuli. If untreated, CH progresses into the decompensatory phase, and which ultimately results in the irreversible heart failure. During this transition phase, an increase in myocardial mass, sarcomeric reorganization, expression of fetal genes, and remodeling of extracellular matrix take place [2].

The extracellular matrix (ECM) of the adult myocardium hosts both cardiomyocytes and interstitial cells in a complex three-dimensional orientation. ECM in addition to mechanical support also serves as a reservoir of growth factors to maintain basal physiology. During myocardial stress, homeostasis of the ECM is perturbed, resulting in systolic and diastolic dysfunctions due to compromised signal transduction [3]. In myocardial remodeling, a fine balance between synthesis and breakdown of ECM components is perturbed. Specifically, matrix metalloproteinase (MMP) activation was reported in various cardiovascular complications [4]. Cardiac fibrosis, an underlying pathophysiological stage in many cardiovascular complications, results due to abnormal ECM deposition [5]. Activation of MMPs and inhibition of tissue inhibitor of matrix metalloproteinases (TIMPs) favors ECM degradation and its accumulation in the myocardium [6].

Several attempts have been made to inhibit ECM remodeling by inhibiting MMPs in the diseased heart and thereby reduce heart failure [7]. But none of the matrix metalloproteinase inhibitors succeed as a drug for heart failure [8]. Therefore, researchers are more interested to explore natural compounds or nutraceutical agents to inhibit ECM remodeling and thus prevent or delay the disease progression. Evidence-based studies in the past have shown the pivotal role of gaseous signaling molecules such as hydrogen sulfide (H_2_S) and sulfur dioxide in mitigating cardiovascular complications [9, 10]. Hence, there is a pressing need to identify novel sulfur molecules to reverse the remodeling of cardiovascular complications. Among all the kinds of vegetables and fruits that are enriched with sulfur-rich compounds, garlic is more promising to show a cardioprotective effect.

Nutraceutical properties of the garlic against various complications are documented in ancient scriptures. Both prophylactic and therapeutic effects of garlic were promising in cardiometabolic complications [11] Despite having myriad beneficial effects of raw garlic, largely, people avoid it because of the gastric disturbing property of garlic due to the presence of allicin.

Earlier, we have reported the promising cardiometabolic properties of garlic [12–14]. During the LC-MS investigation of sulfur compounds in garlic-fed rat serum, we have identified Allylmethylsulfide (AMS) as one of the major garlic-derived metabolites [15]. Similarly, in clinical studies, serum, breast milk, and urine samples also showed the presence of AMS [16, 17]. Pretreatment of AMS ameliorated X-ray-induced inflammation in mouse kidney [18]. It is reported that garlic intervention in various forms ultimately results in the formation of AMS in the physiological system. Therefore, it could be hypothesized that the beneficial effects of garlic such as lipid-lowering, antiatherosclerotic, antidiabetic, antihypertensive, antioxidant, and anticancer are attributed to its active metabolite AMS [19, 20].

Isoproterenol (isoprenaline) is a synthetic nonspecific beta-adrenergic receptor agonist. Sustained release of isoproterenol induces cardiac hypertrophy followed by myocardial remodeling and ultimately leading to heart failure [21]. Biochemical and pathophysiological perturbations due to isoproterenol are very similar to human disease settings and mimic anxiety- and stress-induced cardiac hypertrophy [22]. Hence, isoproterenol-induced cardiac hypertrophy in rodents is considered a suitable model to test the efficacy of novel molecules.

In our previous study, we have reported the antihypertrophic effect of AMS on cardio myoblast [15]. However, the *in vivo* effect of AMS in cardiac hypertrophy and molecular mechanism underlying the beneficial effect, if any, is not explored yet. Therefore, the present study was designed to find the effect of AMS on isoproterenol-induced cardiac hypertrophy and to explore its molecular mechanism mostly focusing on oxidative stress, apoptosis, and alteration of ECM components.

## 2. Material and Methods

### 2.1. Animals and Study Design

Male Sprague Dawley Rats of 200-250-gram weight were procured form the National Institute of Pharmaceutical Education and Research (Mohali, India). All animal studies were performed in accordance with the standard operating procedures of the Translational Health Science and Technology Institute (THSTI) and with Institutional Animal Ethical Committee (IAEC/THSTI/2015-4) approval, Faridabad. Animals were housed in a small animal facility of THSTI, maintained at a 22 ± 2°C temperature, 50 ± 15% relative humidity, and 12 hrs of dark and light cycle.

#### 2.1.1. Safety Study

Fifty-six animals were randomly divided into seven groups (*n* = 8): Group 1 (control), Group 2 (corn oil/vehicle control), Group 3 (garlic 250 mg/kg), Group 4 (AMS 25 mg/kg/day), Group 5 (AMS 50 mg/kg/day), Group 6 (AMS 100 mg/kg/day), and Group 7 (AMS 200 mg/kg/day).

#### 2.1.2. Efficacy Study

Forty animals were randomly divided into five groups (*n* = 8): Group 1 (control), Group 2 (hypertrophy), Group 3 (hypertrophy+25 mg/kg/day), Group 4 (hypertrophy+AMS 50 mg/kg/day), and Group 5 (hypertrophy+enalapril 10 mg/kg/day).

### 2.2. Dosage Information

For safety study, 0.5 ml of virgin corn oil (Group 2), freshly prepared garlic homogenate 250 mg/kg/day along with 0.5 ml of corn oil (Group 3), and AMS 25, 50, 100, and 200 mg/kg (Groups 4, 5, 6, and 7) dissolved in 0.5 ml of corn oil were administered orally for 30 days. For efficacy study, 0.5 ml of corn oil was orally administered as vehicle in three groups (Group 1, Group 2, and Group 5) while AMS (25 and 50 mg/kg/day) dissolved in 0.5 ml was orally administered in two groups (Groups 3 and 4). Group 5 received 10 mg/kg of enalapril orally.

### 2.3. Electrocardiography

Rats were anesthetized in supine position by gaseous anesthesia (isoflurane 2%) coupled with a 100% oxygen supply. The core body temperature of the animal was maintained at 37°C by a controlled heating pad (Homeothermic Blanket Control unit, Harvard Apparatus®). As per the manufacturer's instructions, 15 minutes of ECG was recorded to each animal on Power Lab 26T (ADInstruments, Australia) and the acquired data was analyzed on LabChart 8 software.

### 2.4. Serum Biochemical Parameters

Safety study animals were subjected to retroorbital bleeding under gaseous anesthesia. Blood samples were kept at room temperature for 1 hr followed by centrifugation at 3,000 g for 30 minutes at 4°C. Serum glutamic oxaloacetic transaminase (SGOT), serum glutamic pyruvic transaminase (SGPT), creatinine kinase-myocardium bound (CK-MB), and alkaline phosphatase were measured by a semi autoanalyzer.

### 2.5. Single-Dose Pharmacokinetics of Allylmethylsulfide

To study the pharmacokinetics of AMS, SD rats were used. AMS 100 mg/kg single dose was administered orally, and subsequently, blood samples were collected at 0, 0.25, 0.5, 1, 2, 4, 8, 12, 24, 36, and 48 hrs. Post 30 minutes of sample collection, serum was separated by centrifugation at 600 g for 20 minutes at 4°C and stored at -80°C for further analysis.

#### 2.5.1. Metabolite Extraction

For sample preparation, 100 *μ*l of serum was mixed with 100 *μ*l of LC-MS grade methanol and vortexed for 10 min at room temperature. Further, the sample was incubated on ice for 15 min and centrifuged at 16000 g for 20 min at 4°C. The resultant supernatant was filtered with a 0.2 *μ*m filter.

#### 2.5.2. Metabolomics Measurement

For standard curve, AMS (Cas. No. 10152-76-8, Sigma-Aldrich), AMSO (Cas. No. 21892-75-1, EPTES, Food and Flavor Analytical), and AMSO_2_ (Cas. No. 16215-14-8, Sigma-Aldrich) were spiked in control serum and the earlier mentioned extraction procedure was followed. All data were acquired on the orbitrap fusion mass spectrometer equipped with a heated electrospray ionization (HESI) source. Data were acquired on a positive mode at 120,000 resolution in full-scan MS1. We used a spray voltage of 4000 for positive. Sheath gas and auxiliary gas were set to 42 and 11, respectively. The mass scan range was 50-500 m/z, AGC (automatic gain control) target at 400000 ions, and the maximum injection time was 200 ms for MS. Extracted metabolites were separated on UPLC ultimate 3000 using an HSST3 column (100 × 2.1 mm i.d, 1.9 micrometer, Waters Corporation) maintained at 40°C temperature. Mobile phase A was methanol with 0.1% formic acid, and mobile phase B was water with 0.1% formic acid. The elution gradient is used as follows: 0 min, 1% B; 1 min, 15% B; 4 min, 35% B; 7 min, 95% B; 9 min, 85% B; 10 min, 1% B; and 14 min, 1% B. The flow rate of 0.3 ml/min, and the sample injection volume were 5 microliters.

#### 2.5.3. Data Processing

All acquired data has been processed using Progenesis QI for metabolomics (Waters Corporation) software using the default setting. The untargeted metabolomics workflow of Progenesis QI was used to perform retention time alignment, feature detection, deconvolution, and elemental composition prediction. Identification of metabolites has been done based on the match of accurate mass and the retention time of purchased standards. Relative intensity of the corresponding metabolites has been used for quantification. PKSolver a freely available add-in program for Microsoft Excel was used for pharmacokinetic parameters as mentioned [23].

### 2.6. In Vivo Cardiac Hypertrophy Model

Isoproterenol (~5 mg/kg/day) prepared in 0.001% ascorbic acid solution was delivered by ALZET® osmotic minipump (model #2002) as per the manufacturer's instructions. Briefly, rats were anesthetized with gaseous anesthesia (isoflurane 2%) coupled with a 100% oxygen supply. The core body temperature of the animal was maintained at 37°C by a controlled heating pad (Homeothermic Blanket Control unit, Harvard Apparatus®). Hairs on the dorsal side below the neck are removed by depilatory cream, and a small incision is made to accommodate the sterile minipump charged with isoproterenol solution subcutaneously. Finally, 4-0 silk sutures are used to close the incision. Control animals received osmotic minipump filled with 0.001% ascorbic acid alone. Finally, povidone-iodine ointment was applied until complete wound healing is achieved. Minipump (model 2002) dispenses 0.5 *μ*l per hour for 14 days.

### 2.7. Biochemical Estimation

#### 2.7.1. Lipid Peroxidation

Lipid peroxidation in the myocardium was measured by the protocol described by Ohkawa et al. [24]. Briefly, an equal amount of tissue is homogenized in 10% (*w*/*v*) of ice-cold 0.05 M potassium phosphate buffer (pH 7.4). Each homogenate (0.2 ml) was added to 0.2 ml of 8.1% SDS, 1.5 ml of 20% acetic acid, and 1.5 ml of 0.8% thiobarbituric acid (TBA). Distilled water was added to make up the volume to 4.0 ml, and the solution was kept on water bath maintained at 95°C for 1 hr. Finally, the supernatant was mixed with an equal volume of butanol : pyridine (15 : 1) and centrifuged and the optical density of the organic layer was measured at 532 nm. For quantification of malondialdehyde (MDA) formed in the myocardium, we make a standard curve after putting 1,1,3,3-tetraethoxypropane in different concentrations and mixed with the TBARS as mentioned above.

#### 2.7.2. Reduced Glutathione

Reduced glutathione in the myocardium was measured by Ellman's method [25]. Briefly, an equal amount of heart tissue was homogenized in 10% (*w*/*v*) of ice-cold 0.05 M potassium phosphate buffer (pH 7.4). The resultant homogenate was centrifuged at 15,800 g for 30 minutes at 4°C. To deproteinize, 0.5 ml of the above supernatant was mixed with 0.5 ml of 5% trichloro acetic acid (TCA) and centrifuged at 2,300 g for 10 minutes. The deproteinized 0.5 ml sample is mixed with 0.25 ml of dithio-nitro-benzoic-acid (DTNB) and 1.5 ml of 0.3 M disodium hydrogen phosphate. Finally, the optical density of the mixture was measured at 412 nm. The readout of the sample was normalized by total protein present as measured by the bicinchoninic acid assay method (Thermo Scientific).

#### 2.7.3. Catalase Estimation

Myocardial catalase was estimated by the method as described by Aebi [26]. Briefly, tissue samples were homogenized as mentioned earlier. About 20 *μ*l of tissue supernatant was mixed with 0.5 ml of 50 mM phosphate buffer (pH 7.0), and finally, 0.25 ml of 30 mM H_2_O_2_ is added, and change in the absorbance at 240 nm was recorded for 1.5 minutes with a 15-second interval. Catalase activity is expressed as the decomposition of H_2_O_2_/min/mg of protein.

#### 2.7.4. Superoxide Dismutase

Total superoxide dismutase activity is measured as per the manufacturer's protocol using the Sigma-Aldrich (19160-1KT-F) kit.

### 2.8. Immunoblotting

Approximately 50 mg of myocardial tissue is homogenized in 1 ml of RIPA buffer containing (1x) protease and phosphatase inhibitors. The homogenate is centrifuged at 13,500 g for 20 min at 4°C. Protein concentration in the supernatant is measured by the the bicinchoninic acid assay method (Thermo Scientific). Sample preparation is done in Laemmli buffer using an equal amount of protein. For electrophoresis, protein is resolved on 10% SDS-polyacrylamide gel prepared by the TGX stain-free kit (Bio-Rad). Methanol-activated 0.2 *μ*m pore size (Thermo Scientific) polyvinylidene difluoride (PVDF) membrane is used for protein transfer after electrophoresis. To avoid nonspecific binding of antibodies, membranes are blocked with 5% bovine serum albumin (BSA) prepared in tris-buffered saline (TBS) containing 0.1% Tween 20 for 60 min at room temperature. Primary antibody incubation is done overnight at 4°C. To remove the unbound primary antibody, the membrane is washed with 1x TBST thrice with 5 min interval each.

The specific HRP-labelled secondary antibody is incubated at room temperature for 60 min. Further, the membrane is washed with TBST thrice and finally, the signal is recorded using the Gel Doc XR system (Bio-Rad) with West Dura Pico kit (Thermo Scientific). The following antibodies were used in the study: beta MHC (Abcam; ab50967), MnSOD (Abcam; ab137037), catalase (Abcam; ab52477), caspase 3 (Cell Signaling; 9665), caspase 7 (Cell Signaling; 12827), caspase 9 (Cell Signaling; 9508), MMP2 (Abcam; ab86607), MMP9 (Abcam; ab38898), TIMP3 (Cell Signaling; 5673), and GAPDH antibody (Cell Signaling; 2118). Measured protein expression was normalized to GAPDH as a housekeeping protein.

### 2.9. Gene Expression

Total RNA was isolated from the left ventricular tissue by TRI reagent (Sigma-Aldrich) as per the manufacturer's protocol. The purity and concentration of RNA were measured by a NanoDrop spectrophotometer (Thermo Scientific). Following DNase treatment, reverse transcriptase reaction was performed by SuperScript-III Reverse Transcriptase (Takara, USA) for cDNA synthesis from 2 *μ*g of RNA. Primer sequences for real-time polymerase chain reaction (RT-PCR) were designed from the published sequences available in the public domain. RT-PCR was performed on Roche Light Cycler using the SYBR Green mix (Takara, USA). The data obtained were normalized to RPL32 expression as a reference gene. The following primer sequences were used in the study as mentioned in Table 1.

### 2.10. Histopathology

Immediately after sacrifice of the whole heart, kidney and liver tissues were excised and cleaned with ice-cold PBS to remove blood clot. Histopathology samples were stored in freshly prepared 10% phosphate-buffered formalin. Masson's trichrome and haematoxylin-eosin stains were used to stain 5 *μ*m thick sections prepared from the paraffin block. To examine the histopathological changes, the Nikon Eclipse Ti microscope was used.

### 2.11. Cell Culture and Treatments

The rat cardio myoblast (H9c2) cell line was procured from ATCC® (USA) and was cultured in Dulbecco's Modified Eagle Media, (Cat. No. SH30243.01, HyClone™, GE Life Sciences) containing (4 mM L-glutamine and 45000 mg/L glucose and sodium pyruvate) and supplemented with 10% Fetal Bovine Serum (Cat. No. SH30071.03, HyClone™, GE Life Sciences). Cell culture was maintained at 37°C in a 5% CO_2_ incubator (HERACELL VIOS 160I, Thermo Scientific). For flow cytometry and confocal imaging, 0.2% ethanol was added to the control group. The hypertrophy group was treated with 10 *μ*m isoproterenol (Sigma-Aldrich) along with 0.2% ethanol, and the treatment group received 50 *μ*m AMS along with 10 *μ*m isoproterenol. In every experiment, it is made sure that the final volume of ethanol did not exceed 0.2%. During 72 hrs of the abovementioned treatments, H9c2 cells were grown in DMEM containing 1% FBS with intermittent medium rechange at every 24 hrs.

### 2.12. ROS Detection by Flow Cytometry and Immunostaining

Intracellular ROS is measured by BD FACSCanto™ II (BD Biosciences, US). Briefly, following 72 hrs of earlier mentioned treatments, 10 *μ*m dichlorodihydrofluorescein diacetate (Cat. No. D399, Invitrogen, San Diego, CA) is incubated for 30 minutes at 37°C. Cells were trypsinized with 1x Trypsin-EDTA (Cat. No. CC5027.010L, Cell Clone™) and centrifuged at 210 g for 10 minutes with two times of PBS washing. The results were analyzed by FlowJo™ software for Windows Version V10, Ashland.

For immunostaining, we followed the previously described protocol [27], Briefly, following 72 hrs of earlier mentioned treatment, 10 *μ*m DCFDA was incubated for 30 minutes at 37°C and washed twice with 1x PBS. H9c2 cells on coverslip are fixed with 10% menthol prepared in 1x PBS for 5 minutes at room temperature. Finally, after two times of PBS washing, the coverslips were mounted on a glass slide with mounting media (Cat. No. H-1200, VECTASHIELD, Vector Laboratories, Inc., Burlingame, CA) containing DAPI. Fluorescent images were captured with a FV 3000 (OLYMPUS, Life Science Solutions) laser scanning confocal microscope. The images were analyzed by FIJI (NIH-Image J) software.

### 2.13. Caspase 3/7 Assay in H9c2 Cell

H9c2 cardio myoblasts were grown on a glass coverslip and treated with the abovementioned doses for 72 hrs. Caspase 3/7 green fluorescent reagent (Cat. No. C10423, CellEvent™, Invitrogen) is a four-amino acid peptide (DEVD) attached to a nucleic acid-binding dye with absorption/emission maxima of ~502/530 nm. Activated caspase 3/7 will cleave the DEVD peptide sequence and allow it to bind with the nucleic acid and produce a green signal. Following treatments, 5 *μ*m of caspase 3/7 green detection reagent in complete media is incubated for 30 minutes at 37°C. Finally, the media is removed and washed with 1x PBS. Further, to fix the cells, 4% paraformaldehyde was used for 15 minutes. Coverslips are mounted on a glass slide with mounting media added with nuclei counter stain DAPI and imaged using a FV 3000 machine. For analysis, mean fluorescence in the nuclei region was considered by FIJI software.

### 2.14. MTT Assay

In vitro AMS toxicity is determined by methyl thiazolyl tetrazolium (MTT) assay. Briefly, cardio myoblasts (10,000 cells/well) were seeded in each 96 well plate to attain a 60-70% confluency. Further, AMS dose range (100 nm to 500 *μ*m) in ethanol was treated for 24 hrs and then incubated with 1 mg/ml of MTT for 4 hrs in complete media. During MTT incubation, live cells form purple-colored formazan crystals. Unused MTT in the supernatant was removed, and media was replaced with 50 *μ*l of dimethyl sulfoxide (DMSO), and then incubated for 30 minutes at 37°C to dissolve the crystals. Finally, absorbance at 570 nm was recorded on a microplate reader (Molecular Devices). The effect of AMS on H9c2 proliferation was expressed as relative percentage viability. Percent viability = (OD of treatment/OD of control)∗100.

### 2.15. Statistical Analysis

Data in the present study is reported as the mean ± standard error of the mean (S.E.M). Mean differences among the study group were analyzed by one-way analysis of variance (ANOVA), followed by the Bonferroni multiple comparison test. A significance level is assumed if *p* < 0.05. Statistical analysis was performed in GraphPad Prism 8.2.1 (279) (Graph Pad Software Inc., San Diego, CA, USA).

## 3. Results

### 3.1. Allylmethylsulfide Is Rapidly Metabolized into Allylmethylsulfoxide and Allylmethylsulfone

**Figure 1:**
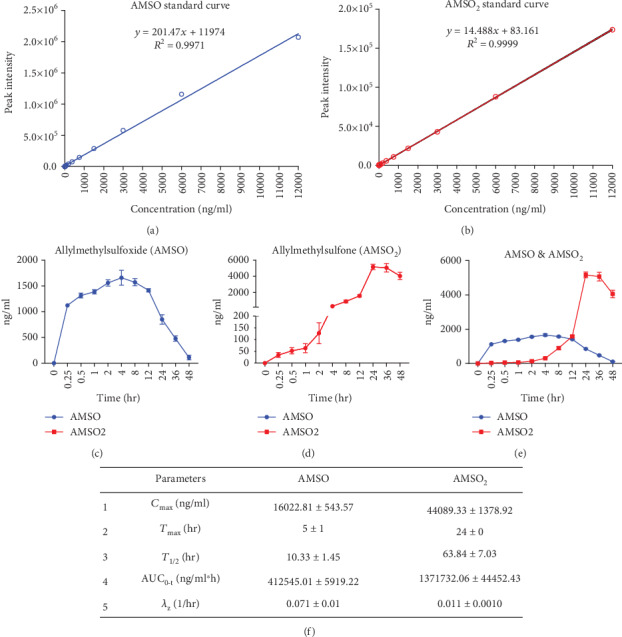
Effect of Allylmethylsulfide on pharmacokinetic parameters. (a) Standard curve of Allylmethylsulfoxide (AMSO). (b) Standard curve of Allylmethylsulfone (AMSO_2_). (c, d) Concentration vs time graph of AMSO and AMSO_2_. (e) Cumulative representation of graphs c and d. (f) Pharmacokinetic parameters of AMSO and AMSO_2_. Maximum serum concentration (*C*_max_), time taken to reach maximum serum concentration (*T*_max_), elimination half-life (*T*_1/2_), area under curve from time “0” to time “*t*” (AUC_0-t_), and terminal elimination rate constant (*λ*_z_). Data were represented as mean ± SEM (*n* = 4).

To study the pharmacokinetics of AMS, a single dose of 100 mg/kg was administered orally. We have observed that AMS is rapidly metabolized into AMSO and AMSO_2_ in the physiological system. To measure the exact concentration of AMSO and AMSO_2_ in the serum, peak intensity of the metabolites was extrapolated to the standard curve of the same, respectively (Figures 1(a) and 1(b)). With time, the concentration of AMSO and AMSO_2_ is gradually increased and showed peak at 5 hrs and 24 hrs, respectively (Figures 1(c)–1(e)). Pharmacokinetic parameters, derived from concentration vs time curve by PKsolver, suggests that half-life of AMSO is less (10.33 hrs) compared to AMSO_2_ (63.84 hrs) (Figure 1(f)). The *C*_max_ for AMSO and AMSO_2_ are 16022.81 and 44089.33 ng/ml, respectively (Figure 1(f)).

### 3.2. Allylmethylsulfide Is Safe in Rats on Chronic Administration

**Figure 2:**
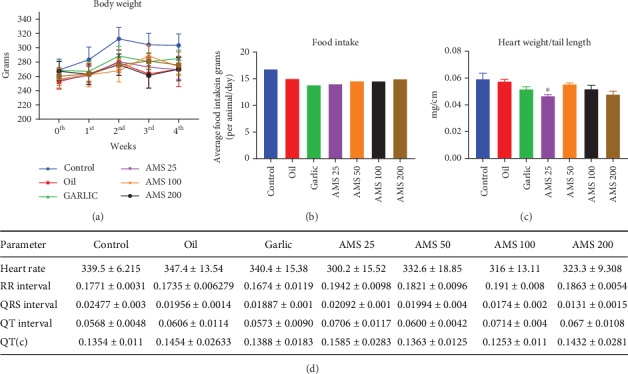
Effect of Allylmethylsulfide on the (a) body weight, (b) food intake, (c) heart weight to tail length ratio, and (d) electrocardiogram (ECG) parameters. Data were represented as mean ± SEM, *n* = 5 for ECG and *n* = 8 for other parameters. ^∗^*p* < 0.05 vs control.

To evaluate the effect of chronic administration of AMS, we have measured the body weight of the animals at every week from the beginning of AMS intervention till 4 weeks. We did not observe any significant change in the body weight in any of our treatment groups (Figure 2(a)). Similarly, food consumed by each rat per day (normalized with the number of animals per cage) did not show a major difference between the groups (Figure 2(b)). Except for AMS 25 mg/kg dose, there was no significant decrease in the heart weigh to tail length ratio compared with other doses (Figure 2(c)). Further, to study the impact to AMS on electrical conduction of the myocardium, we have performed electrocardiogram (ECG), at the end of 30 days of the treatment. We did not observe significant differences in any of the ECG parameters (Figures 2(d)).

### 3.3. Histopathology and Serum Biomarkers of Vital Organs Remained Normal on Chronic Administration of Allylmethylsulfide

**Figure 3:**
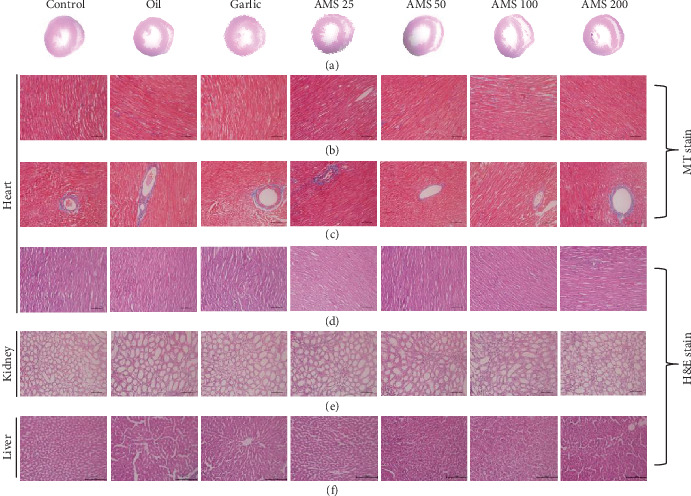
Effect of AMS on histopathology. (a) Transverse section of the heart representing the ventricular diameter. (b, c) Masson's trichrome staining of the heart tissue representing interstitial and perivascular fibrosis, respectively. (d–f) Haematoxylin and eosin stain of the heart, kidney, and liver.

To study the effect of AMS on histopathology of vital organs such as the heart, liver, and kidney, we have stored the tissues immediately after the euthanasia and stained it with MT and H&E stain. We did not observe any structural difference between any of the treatment groups (Figures 3(a)-3(f)). Before sacrifice, we have collected the serum and measured biomarkers for liver and heart injury. Here, we did not observe a significant difference in serum glutamic oxaloacetic transaminase (SGOT) (Figure S1 (a)), serum glutamic pyruvic transaminase (SGPT) (Figures S1 (b)), creatinine kinase-myocardium bound (CK-MB) (Figure S1 (c)), and alkaline phosphatase (Figure S1 (d)) levels among any of the AMS treatment groups.

### 3.4. Allylmethylsulfide Ameliorates Isoproterenol-Induced Cardiac Hypertrophy

**Figure 4:**
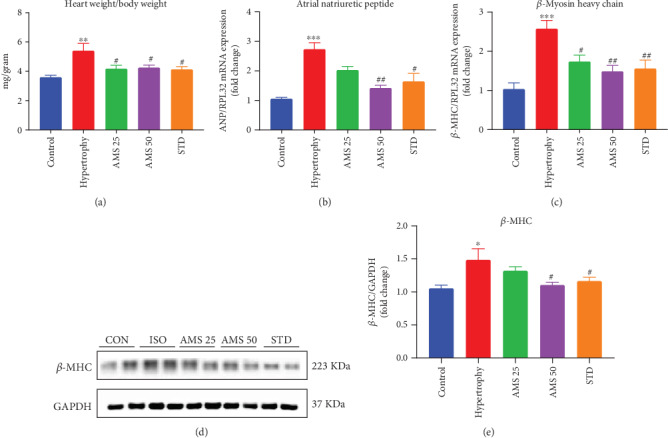
Effect of AMS on cardiac hypertrophy markers. (a) Heart weight to body weight ratio. (b) mRNA expression of atrial natriuretic peptide (ANP). (c) mRNA expression of beta myosin heavy chain (*β*-MHC). (d) Representative western blot images of *β*-MHC and GAPDH. (e) Densitometric analysis of *β*-MHC. mRNA expression data were normalized to the expression of the reference gene, RPL32. Protein expression data were normalized with the reference protein expression, GAPDH. Data were expressed as mean ± SEM, (*n* = 5 for mRNA expression and *n* = 4 for protein expression). ^∗^*p* < 0.05, ^∗∗^*p* < 0.01, ^∗∗∗^*p* < 0.001 vs control and ^#^*p* < 0.05, ^##^*p* < 0.01 vs hypertrophy.

Isoproterenol-induced cardiac hypertrophy was measured by the heart weight to body weight (HW/BW) ratio at the end of 14 days of AMS cotreatment. We have noticed there is a significant increase in the HW/BW ratio in the diseased group, while with the AMS and enalapril treatment, it was reduced (Figure 4(a)). mRNA expression of the fetal genes such as atrial natriuretic peptide (ANP) (Figure 4(b)) and beta myosin heavy chain (*β*-MHC) (Figure 4(c)) was significantly reduced with the AMS and enalapril treatment in the myocardium. Further, we have measured the protein expression of *β*-MHC and noticed that AMS (50 mg/kg/day) and enalapril significantly reduced it in the hypertrophied heart (Figure 4(e))

### 3.5. Allylmethylsulfide Reduced Lipid Peroxidation and Improved Endogenous Antioxidants in Cardiac Hypertrophy

**Figure 5:**
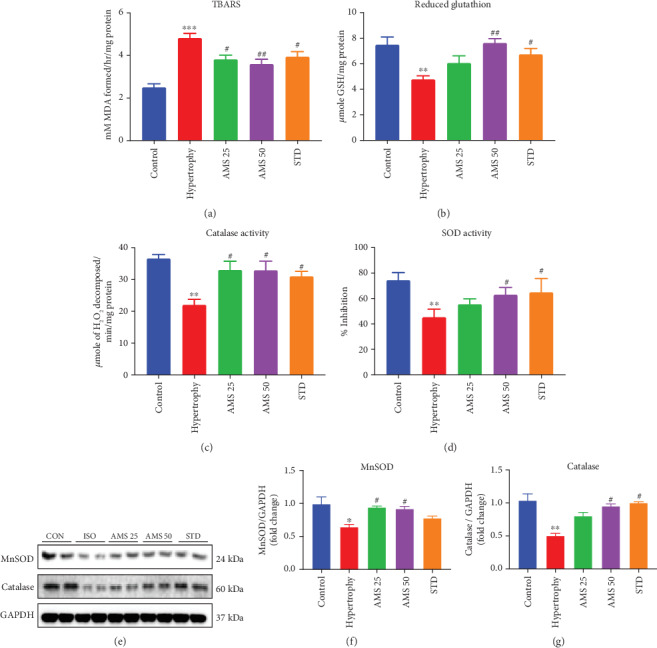
Effect of AMS on biochemical parameters and endogenous antioxidants. (a) Thiobarbituric acid reactive substances (TBARS). (b) Reduced glutathione (GSH). (c) Catalase activity. (d) Super oxide dismutase activity (SOD). (e) Representative western blot images of MnSOD, catalase, and GAPDH. (f) Densitometric analysis of manganese superoxide dismutase (MnSOD). (g) Densitometric analysis of catalase expression. Protein expression data were normalized with the reference protein expression, GAPDH. Data were expressed as mean ± SEM, (*n* = 5 for biochemical parameters and *n* = 4 for protein expression). ^∗^*p* < 0.05, ^∗∗^*p* < 0.01, and ^∗∗∗^*p* < 0.001 vs control group and ^#^*p* < 0.05, ^##^*p* < 0.01 vs hypertrophy.

We next decided to study the effect of AMS on lipid peroxidation and endogenous antioxidants. Isoproterenol induced a significant increase in the MDA levels as measured by TBARS, and with the AMS (25 and 50 mg/kg/day) and enalapril treatment, it was reduced significantly (Figure 5(a)). The enzyme levels of endogenous antioxidant such as glutathione (GSH) were reduced with the isoproterenol treatment, and with AMS (50 mg/kg/day) and enalapril, it was preserved (Figure 5(b)). Similarly, decreased catalase and superoxide dismutase (SOD) activity in isoproterenol-treated hearts was improved with the AMS and enalapril treatment (Figures 5(c) and 5(d)). To further check the protein expression of catalase and MnSOD, we did immunoblot analysis. We observed that AMS and enalapril treatment increased both of their protein levels in the hypertrophic heart (Figures 5(f) and 5(g)).

### 3.6. Allylmethylsulfide Ameliorated Reactive Oxygen Species in Isoproterenol-Treated Cardio Myoblast

To corroborate the results of the *in vivo* study, we have treated H9c2 cardio myoblast for 72 hrs. With isoproterenol (10 *μ*m) and cotreated with AMS (50 *μ*m). Further, we have measured the ROS generation by flow cytometry (Figures S3 (A-D)) and immunofluorescence (Figures S4 (a)-S4(d)). Similar to our *in vivo* study, we have observed that AMS cotreatment reduced the isoproterenol-induced ROS generation. These two independent experiments confirmed that AMS has a property to reduce the ROS production. We have also checked the viability of the H9c2 cells post 24 hrs treatment with AMS and did not find any significant cytotoxicity (Figure S2).

### 3.7. Allylmethylsulfide Prevented Extracellular Matrix Damage by Matrix Metalloproteinases

**Figure 6:**
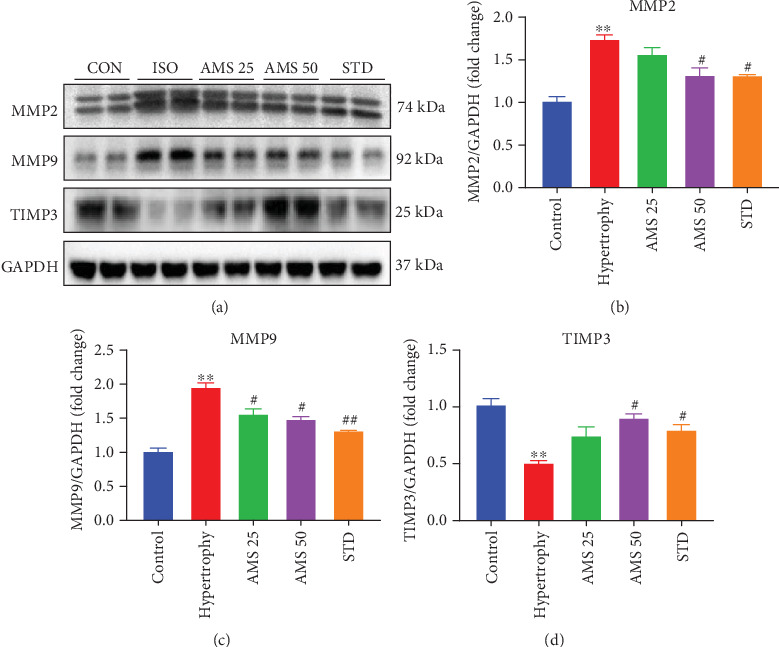
Effect of AMS on extracellular matrix components. (a) Representative western blot images of caspase matrix metalloproteinases (MMPs 2 and 9), tissue inhibitor of matrix metalloproteinase 3 (TIMP3), and GAPDH. (b, c) Densitometric analysis of MMPs 2 and 9. (d) Densitometric analysis of TIMP3. Protein expression data were normalized with the reference protein expression, GAPDH. Data expressed as mean ± SEM, (*n* = 4). ^∗∗^*p* < 0.01, ^∗∗∗^*p* < 0.001 vs control group and ^#^*p* < 0.05, ^##^*p* < 0.01 vs hypertrophy.

During isoproterenol-induced cardiac hypertrophy, homeostasis of extracellular matrix is perturbed and may result in the activation of matrix metalloproteinases (MMPs). We have observed a significant increase in the protein expression of MMP2 in the hypertrophic group, and with AMS (50 mg/kg/day) and enalapril treatment, it was reduced significantly (Figures 6(b)). Similarly, MMP9 expression was also reduced with the AMS (25 and 50 mg/kg/day) and enalapril treatment (Figure 6(c)). Tissue inhibitor of matrix metalloproteinases (TIMP) modulates the activity of MMPs. We have noticed a reduction of TIMP3 in the isoproterenol-treated heart. However, AMS (50 mg/kg/day) and enalapril pretreatment preserved the TIMP3 expression in the isoproterenol-treated heart (Figure 6(d)).

### 3.8. Allylmethylsulfide Reduced Apoptosis in the Isoproterenol-Induced Hypertrophic Heart

**Figure 7:**
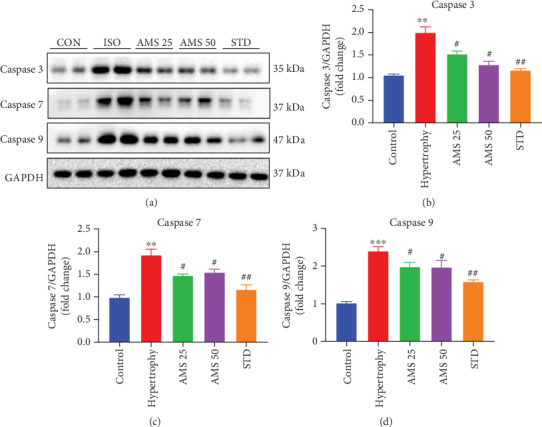
Effect of AMS on caspases. (a) Representative western blot images of caspase 3, caspase 7, caspase 9, and GAPDH. (b–d) Densitometric analysis of caspases 3, 7, and 9. Protein expression data were normalized with the reference protein expression, GAPDH. Data were expressed as mean ± SEM, (*n* = 4). ^∗∗^*p* < 0.01, ^∗∗∗^*p* < 0.001 vs control group and ^#^*p* < 0.05, ^##^*p* < 0.01 vs hypertrophy.

We have studied the protein expression of proapoptotic caspases in the hypertrophic heart. We have observed that isoproterenol-induced hypertrophic hearts showed increased expression of caspase 3 (Figure 7(b)), caspase 7 (Figure 7(c)), and caspase 9 (Figure 7(d)), and the same were significantly reduced with the AMS (25 and 50 mg/kg/day) and enalapril treatment.

### 3.9. Allylmethylsulfide Reduced Apoptosis Signal in Isoproterenol-Treated Cardio Myoblast

**Figure 8:**
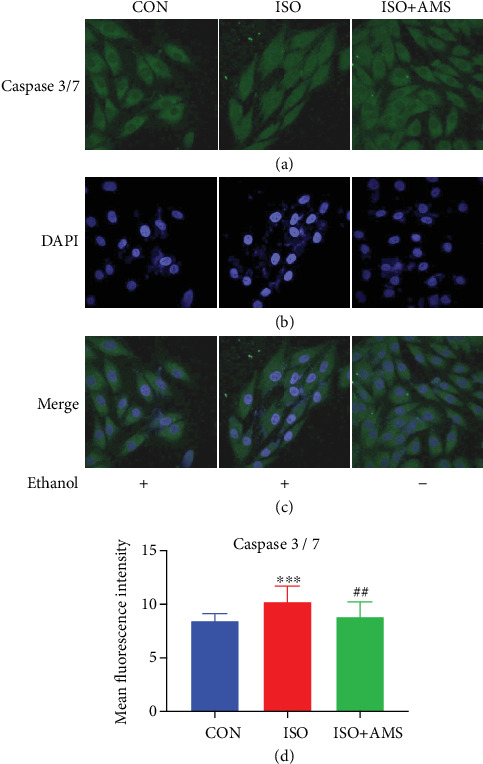
Effect of AMS on caspase 3/7 expression in H9c2 cardio myoblast (a) Representative confocal images of caspase 3/7 expression in the nucleus. (b) Nuclear staining by DAPI. (c) Merged images of DAPI and caspase 3/7. (d) Representative bar graph of mean fluorescence intensity. Data were expressed as mean ± SEM, (*n* = 100 cells/group). ^∗∗∗^*p* < 0.001 vs control (CON) group and ^##^*p* < 0.01 vs isoproterenol (ISO).

To corroborate our in vivo finding, we decided to check the effect of AMS on the nuclear expression of caspase 3/7 in isoproterenol-treated H9c2 cells. We have noticed that there was a significant increase in the green fluorescence of caspase 3/7 in the nuclear portion of isoproterenol-treated cells. However, these signals were reduced in the AMS-cotreated isoproterenol cells (Figures 8(a)-8(d))

### 3.10. Allylmethylsulfide Reduced Fibrosis in the Hypertrophic Heart

**Figure 9:**
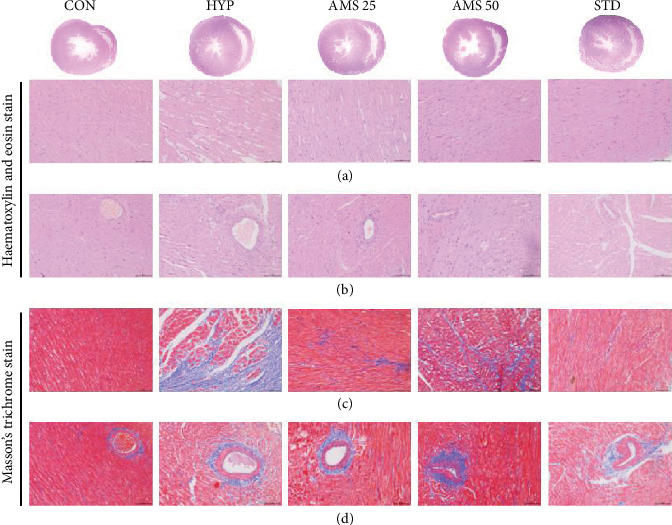
Effect of AMS on isoproterenol-induced hypertrophic heart. (a) Gross transverse section of the heart representing ventricular diameters. (b, c) Haematoxylin and eosin staining of the heart representing neutrophil infiltration in interstitial and perivascular, respectively. (d, e) Masson's trichrome staining of the heart representing interstitial and perivascular fibrosis.

We did histopathology study to check the effect of increased protein expression of matrix metalloproteinases on fibrosis. Gross investigation of the left ventricular diameter showed the presence of hypertrophy in the isoproterenol group; however, with AMS treatment, we have seen a decrease in the diameter and muscle thickness (Figure 9(a)). To check the fibrosis, we have stained the heart sections with MT staining. Interstitial and perivascular fibrosis were prominent in the isoproterenol-treated heart. However, a reduction of fibrosis was observed with AMS and enalapril treatment (Figures 9(d) and 9(e)). H&E staining showed a presence of high neutrophil infiltration in the isoproterenol-treated heart. However, AMS and enalapril treatment reduced the extent of neutrophil infiltration (Figures 9(b) and 9(c)).

## 4. Discussion

Both prophylactic and therapeutic effects of garlic in the past have documented numerous promising results. Previously, we have identified that AMS is an active metabolite of garlic and showed a reduction in the cell size *in vitro* [15]. Pretreatment of AMS ameliorated inflammation in mouse kidney by downregulation of NF-*κ*B signaling [18]. However, the safety and efficacy dose of the AMS, particularly in the cardiac hypertrophy model, are not explored yet. In the current study, we have explored the safety of chronic administration of AMS in rats, single-dose pharmacokinetics, and efficacy of AMS in an isoproterenol-induced cardiac hypertrophy model. Chronic intervention of AMS for 30 days did not show any significant difference in the body weight and food intake. However, the heart weight to tail length ratio was slightly lower with only an AMS 25 mg/kg dose. Although, at present, we do not know the reason for this change, we have performed ECG to further evaluate any cardiac abnormalities. ECG parameters did not show any significant difference within the dose range. QT prolongation, an important parameter of drug toxicity, did not alter with any of the doses. Next, we checked the effect of AMS on tissue biomarkers in the blood. We have measured SGOT, SGPT, CK-MB, and alkaline phosphatase in the serum at the end of the study. Any damage in the liver can be diagnosed by high SGOT and SGPT levels in the blood [28]. Alkaline phosphatase is present in most of the tissues; however, the bones and liver have the highest amount. The estimation of CK-MB provides information on cardiac muscles [29]. These biomarkers are leaked out from their respective localization into the blood during tissue damage. We did not observe any significant difference in these biomarkers with our treatments. To corroborate these results, we have checked for the histopathology of the heart, liver, and kidney. However, we did not observe any structural differences in the morphology of these vital organs. Single-dose pharmacokinetics suggest that AMS is well-absorbed through an oral route and rapidly metabolized into AMSO and then subsequently to AMSO_2_. Presence of AMSO and AMSO_2_ in the serum for a longer duration may allow a single dose of AMS for an efficacy purpose. Interestingly, we did not observe mortality in any of our treatment groups, these results proved that AMS is a safe molecule in rats. Further, in a concentration range from 0.1 to 500 *μ*m of AMS, we did not observe cytotoxicity in rat cardio myoblast (H9c2) cells.

Furthermore, after safety and pharmacokinetic studies, we looked the effect of AMS in the rodent model of cardiac hypertrophy. Isoproterenol-induced cardiac hypertrophy upregulates mRNA expression of fetal genes (ANP and *β*-MHC) [30]. In general, they are highly expressed during the early stages of heart development and later remain constitutively expressed in the mature heart. We have observed that the heart weight to body weight ratio, an important parameter of cardiac hypertrophy in animals, and fetal gene expression in heart were significantly reduced in the AMS and enalapril-treated animals. Isoproterenol-induced cardiac hypertrophy is associated with reduced antioxidants and excessive ROS generation. ROS interacts with cellular components and results in lipid peroxidation [31]. Our data showed that there is a significant increase of malondialdehyde (MDA) in hypertrophic hearts, and however, with AMS and enalapril treatment, it was reduced. Antioxidants such as superoxide dismutase (SOD), catalase, glutathione, and glutathione peroxidase play an important role in maintaining the physiological levels of ROS [32]. We have observed decreased activity of antioxidant enzymes in hypertrophic hearts, and however, with AMS and enalapril intervention, it was significantly preserved. We have also found similar results in the protein expression of MnSOD and catalase with AMS and enalapril treatment.

We next thought to corroborate the *in vivo* findings of oxidative stress with the cellular hypertrophy model. To explore the underlying protective effect of AMS, we have studied the ROS generation in H9c2 cells. During myocardial stress, excessive ROS production and a compromised antioxidant effect leads to pathological cardiac hypertrophy and ultimately progresses into heart failure [33]. Recent reports suggest that targeting ROS generation could be a better alternative to ameliorate cardiac hypertrophy [34]. In our study, the immunofluorescence and flow cytometric analysis suggest that AMS cotreatment significantly reduced ROS generation in isoproterenol-treated cardio myoblast.

Fibrosis develops due to excessive accumulation of collagen and other ECM components by the differentiation of fibroblast into myofibroblast. Based on the nature of pathological insult, the three types of cardiac fibrosis that develop are reactive interstitial fibrosis, infiltrative interstitial fibrosis, and replacement fibrosis [35]. In our study, the replacement of the myocardium with the fibrous scar was observed in the isoproterenol-induced hypertrophy hearts. However, we have observed a significant reduction of cardiac fibrosis with the treatment of both AMS and enalapril.

Cardiac fibroblast plays a pivotal role in the ECM homeostasis. The major components of ECM include collagen I, collagen III, fibronectin, laminin, and elastin. ECM maintains the structural integrity of cardio myocytes, fibroblast, and coronary arteries within the myocardium and also maintains the electrical signal conduction for rhythmic contractility of the heart. The integrity of the ECM components is mainly regulated by matrix metalloproteinases (MMPs) and their tissue inhibitors (TIMPs) produced by fibroblasts [36]. MMP2 and MMP9 are the important enzymes involved in the degradation of ECM and are involved in various cardiac complications [37]. AMS and enalapril treatment preserved a significant increase in the protein expression of MMP2 and MMP9 in the isoproterenol-induced hypertrophic heart. TIMPs have an important role in preventing the proteolytic degradation of ECM by MMPs. The lack of TIMP3 has resulted in many cardiovascular complications [38]. In our study, we have noticed AMS and enalapril improved the protein expression of TIMP3 in hypertrophied hearts.

Cardiac fibrosis resulting from MMP expression was observed in the isoproterenol-induced hypertrophic heart and may lead to cardiomyocyte death, i.e., apoptosis. To investigate the effect of AMS on apoptosis, we have studied the protein expression of caspases. Pathological enlargement of the myocardium with isoproterenol results in activation of programmed cell death, compromised contractile function, and eventually heart failure [39]. Besides apoptosis, caspases play an important role in cardiac inflammation. There are evidence-based studies suggesting the role of caspase 3 and caspase 9 in both cellular and animal models of cardiac hypertrophy [40]. The use of caspase 3 inhibitors holds a promising role in reducing cardiac remodeling [41]. We have observed that caspase 3, caspase 7, and caspase 9 protein expressions were significantly increased in hypertrophic hearts; however, with AMS and enalapril, these expressions were reduced. Nuclear expression of caspase 3/7 is a sign of apoptotic induction in cells due to various underlying pathological insults [42]. We have noticed a significant increase in the nuclear expression of caspase 3/7 in isoproterenol-treated H9c2 cells. However, it was reduced with the AMS cotreatment. The data obtained from *in vitro* and *in vivo* studies confirmed that AMS showed some of its beneficial effects in hypertrophic conditions through protection from apoptosis.

## 5. Conclusion

In the present study, we have demonstrated that AMS is a safe molecule in rats. The pharmacokinetic study showed that AMS results into two stable metabolites, i.e., AMSO and AMSO_2_ in the physiological system. AMS reduced cardiac hypertrophy markers such as fetal gene expression and improved endogenous antioxidants. Isoproterenol-induced cardiac fibrosis and dysregulated ECM deposition in the myocardium were reduced with AMS and enalapril treatment. The only limitation of the efficacy study is that we could not measure the functional parameters of the heart by echocardiography. Overall, our study confirms that AMS is a safe and efficacious molecule for the prevention of cardiac hypertrophy and associated remodeling.

## Figures and Tables

**Table 1:** Primers used in RT-PCR analysis.

Gene	Forward primer	Reverse primer
ANP	AGCGAGCAGACCGATGAAGG	AGCCCTCAGTTTGCTTTTCA
Beta MHC	TGGAGCTGATGCACCTGTAG	ACTTCGTCTCATTGGGGATG
RPL32	AGATTCAAGGGCCAGATCCT	CGATGGCTTTTCGGTTCTTA

## Data Availability

The data used to support the findings of this study are available from the corresponding author upon request.

## Supplementary Materials

Supplementary Materials Figure S1: effect of Allylmethylsulfide on serum biochemical parameters. (a) Serum glutamic oxaloacetic transaminase (SGOT). (b) Serum glutamic pyruvic transaminase (SGPT). (c) Creatinine kinase-myocardium bound (CK-MB). (d) Alkaline phosphatase. Data are represented as mean ± SEM (*n* = 4). Figure S2: effect of Allylmethylsulfide on cardio myoblast viability. Post 24 hrs of AMS treatment with a dose range from 0.1 to 500 *μ*m, the fluorescence was analyzed. Figure S3: effect of Allylmethylsulfide on reactive oxygen species (ROS) production in H9c2 cardio myoblast. (a) Representative histogram of control. (b) Representative histogram of isoproterenol. (c) Representative histogram of isoproterenol cotreated with AMS. (d) Representative bar graph of percentage of Alexa Fluor 488-positive cells. Data are expressed as mean ± SEM, ^∗∗∗^*p* < 0.001 vs control (CON) group and ^##^*p* < 0.01 vs isoproterenol (ISO). Figure S4: effect of Allylmethylsulfide on reactive oxygen species (ROS) production in H9c2 cardio myoblast. (a) Representative confocal images of DCFDA. (b) Nuclear staining by DAPI. (c) Merged images of DAPI and DCFDA. (d) Representative bar graph of mean fluorescence intensity. Data are expressed as mean ± SEM, (*n* = 100 cells/group). ^∗∗∗∗^*p* < 0.0001 vs control (CON) group and ^###^*p* < 0.001 vs isoproterenol (ISO).
