# Supplementary material for: Allylmethylsulfide, a Sulfur Compound Derived from Garlic, Attenuates Isoproterenol-Induced Cardiac Hypertrophy in Rats
Source: Oxid Med Cell Longev. 2020 Jun 11;2020:7856318. doi: 10.1155/2020/7856318 (PMC7306095; doi:10.1155/2020/7856318)
Supplement: Supplementary Materials — Figure S1: effect of Allylmethylsulfide on serum biochemical parameters. (a) Serum glutamic oxaloacetic transaminase (SGOT). (b) Serum glutamic pyruvic transaminase (SGPT). (c) Creatinine kinase-myocardium bound (CK-MB). (d) Alkaline phosphatase. Data are represented as mean ± SEM (n = 4). Figure S2: effect of Allylmethylsulfide on cardio myoblast viability. Post 24 hrs of AMS treatment with a dose range from 0.1 to 500 μm, the fluorescence was analyzed. Figure S3: effect of Allylmethylsulfide on reactive oxygen species (ROS) production in H9c2 cardio myoblast. (a) Representative histogram of control. (b) Representative histogram of isoproterenol. (c) Representative histogram of isoproterenol cotreated with AMS. (d) Representative bar graph of percentage of Alexa Fluor 488-positive cells. Data are expressed as mean ± SEM, ∗∗∗p < 0.001 vs control (CON) group and ##p < 0.01 vs isoproterenol (ISO). Figure S4: effect of Allylmethylsulfide on reactive oxygen species (ROS) production in H9c2 cardio myoblast. (a) Representative confocal images of DCFDA. (b) Nuclear staining by DAPI. (c) Merged images of DAPI and DCFDA. (d) Representative bar graph of mean fluorescence intensity. Data are expressed as mean ± SEM, (n = 100 cells/group). ∗∗∗∗p < 0.0001 vs control (CON) group and ###p < 0.001 vs isoproterenol (ISO). [file 7856318.f1.pptx]

## Slide 1
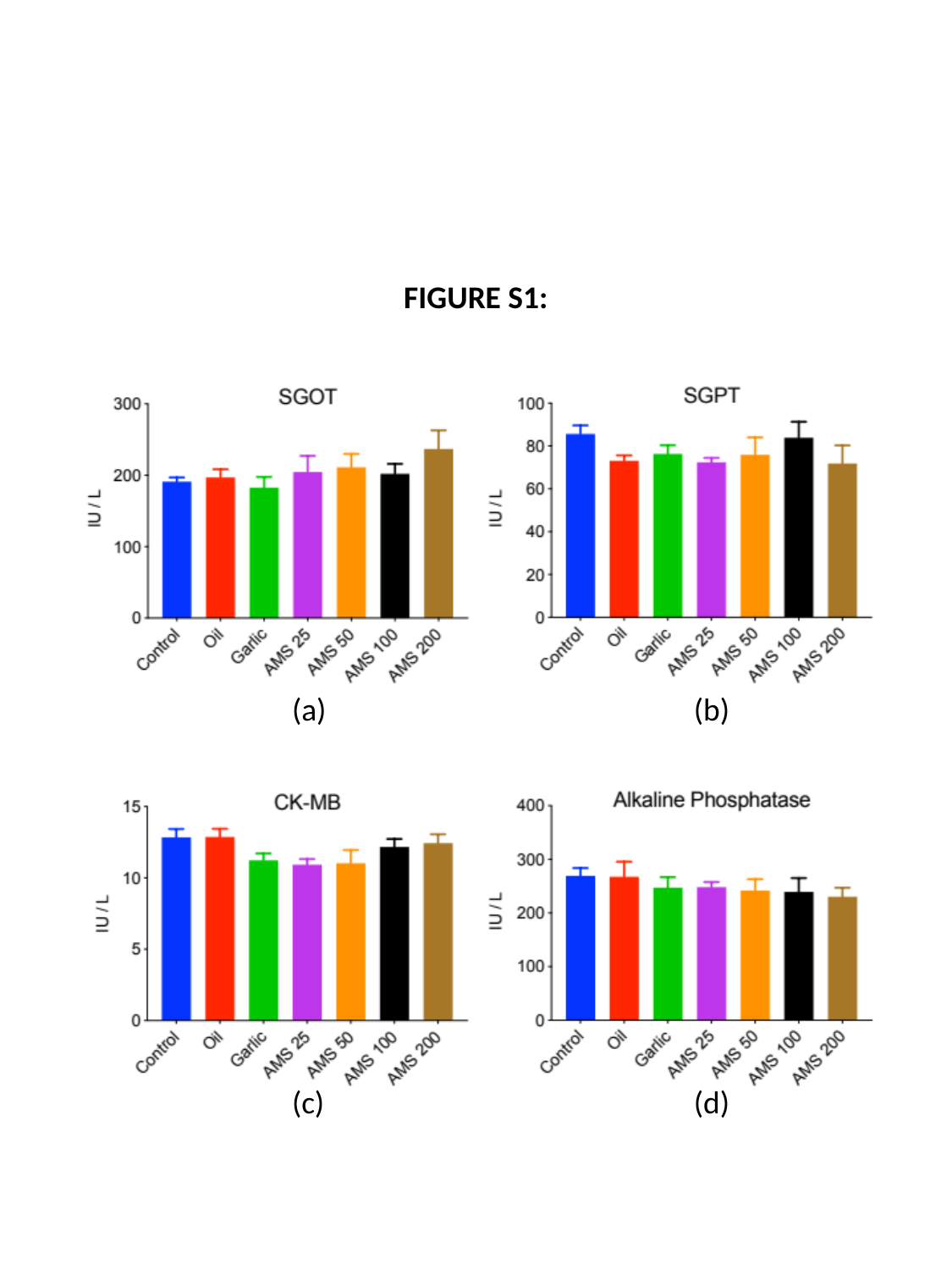

FIGURE S1:
(a)
(b)
(c)
(d)

## Slide 2
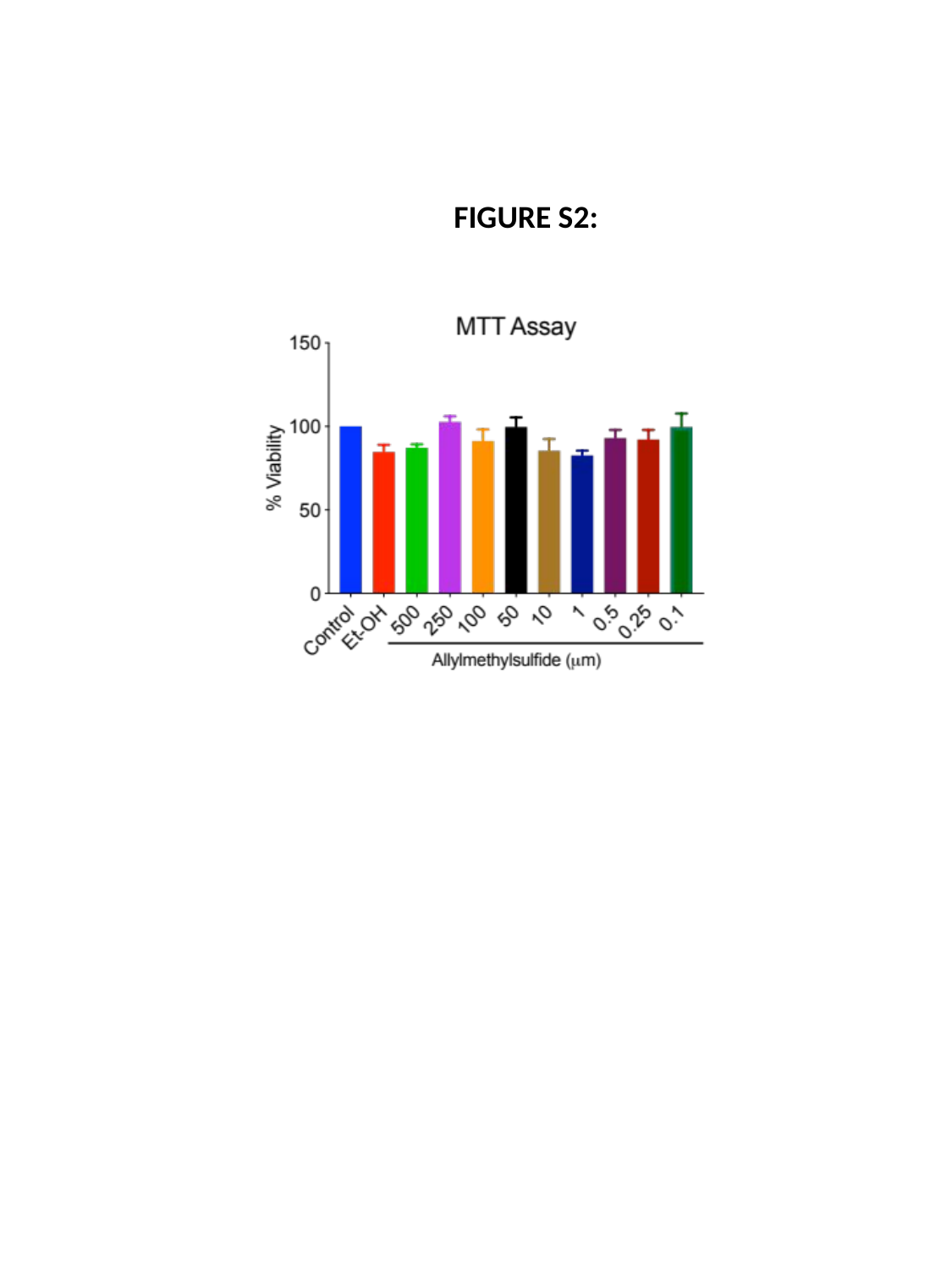

FIGURE S2:

## Slide 3
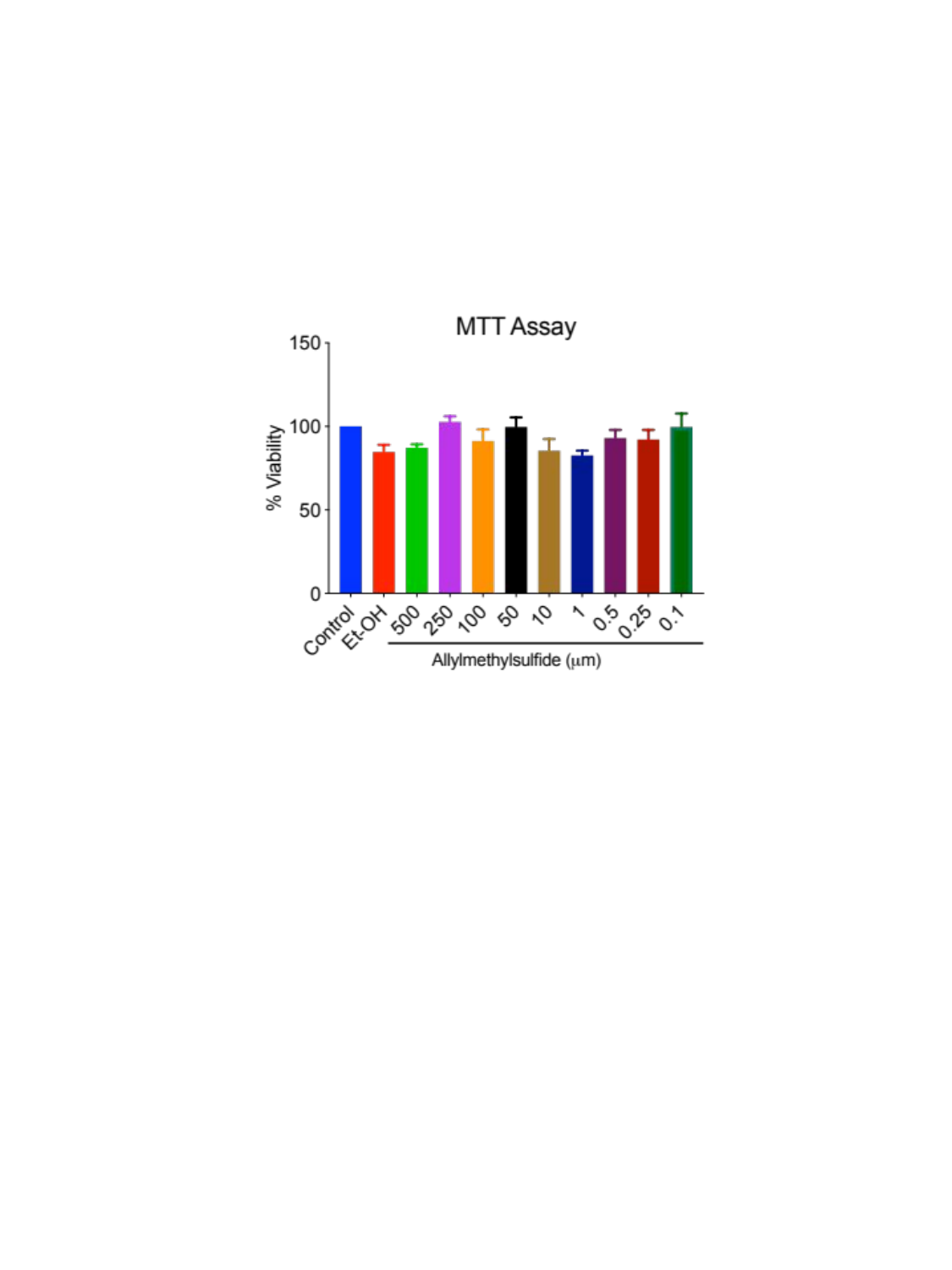

## Slide 4
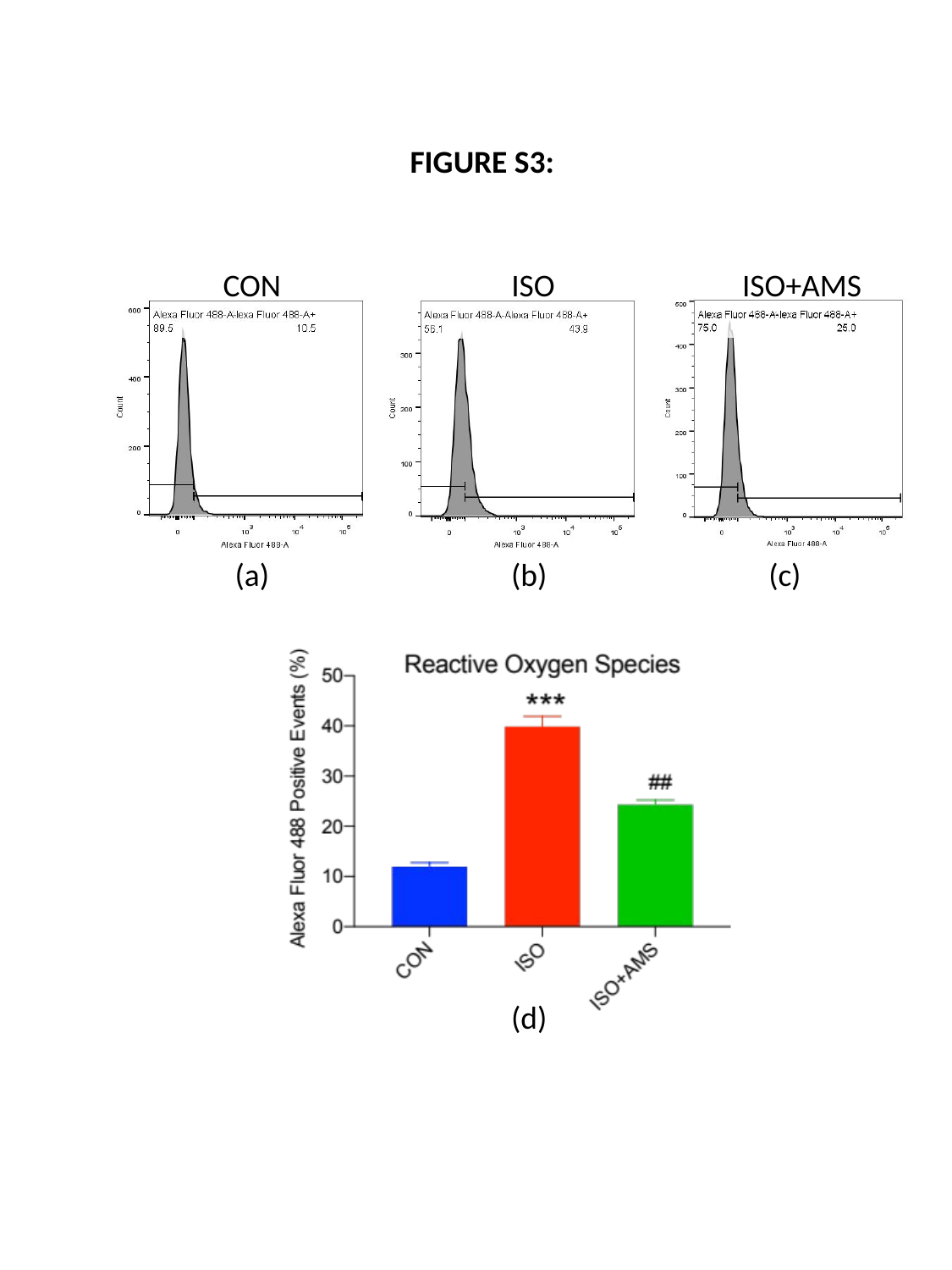

FIGURE S3:
CON
ISO+AMS
ISO
(b)
(c)
(a)
(d)

## Slide 5
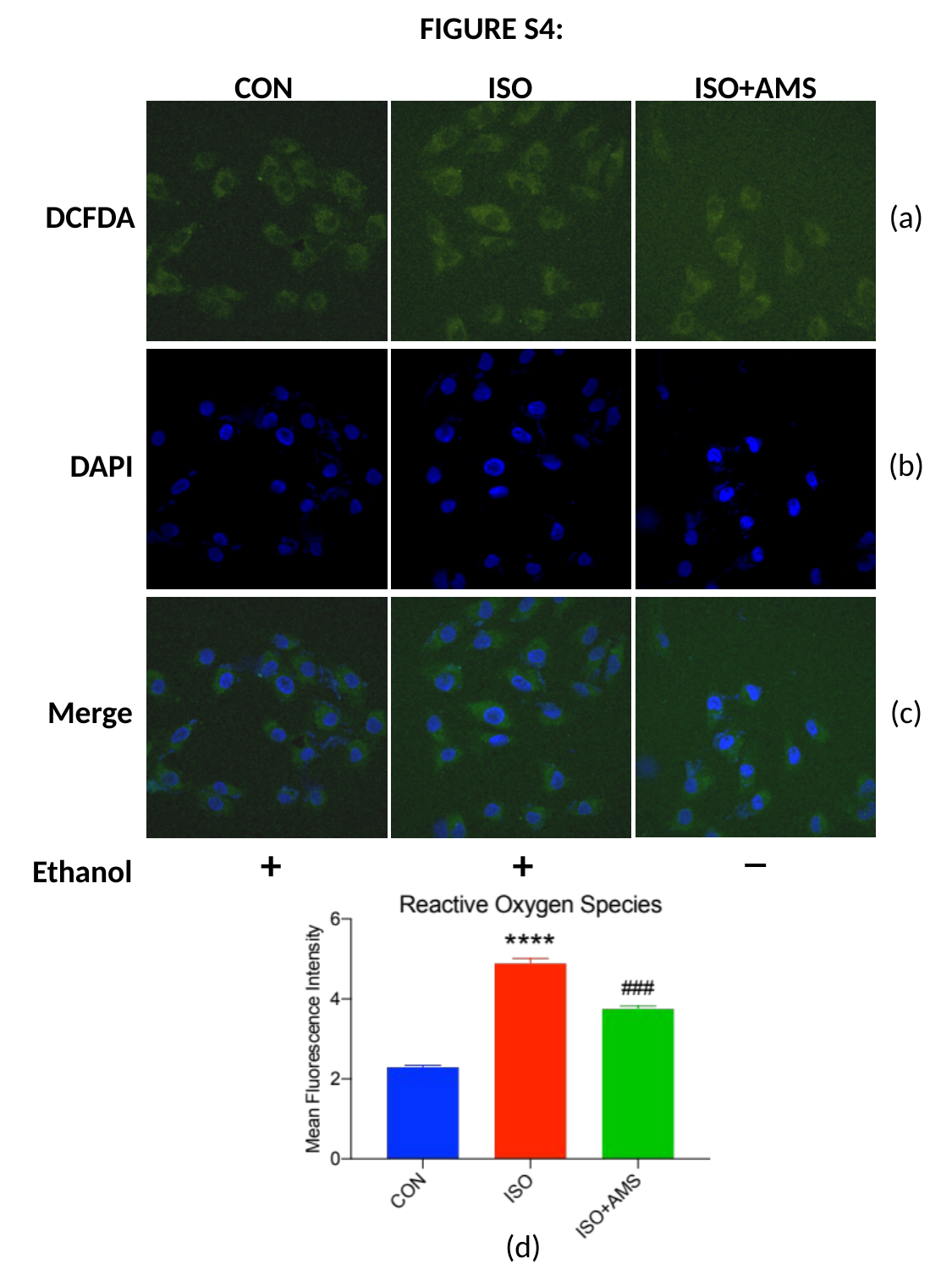

FIGURE S4:
CON
ISO
ISO+AMS
DCFDA
(a)
(b)
DAPI
Merge
(c)
_
+
+
Ethanol
(d)
